# Sodium Glucose Co-Transporter 2 Inhibitors Ameliorate Endothelium Barrier Dysfunction Induced by Cyclic Stretch through Inhibition of Reactive Oxygen Species

**DOI:** 10.3390/ijms22116044

**Published:** 2021-06-03

**Authors:** Xiaoling Li, Gregor Römer, Raphaela P. Kerindongo, Jeroen Hermanides, Martin Albrecht, Markus W. Hollmann, Coert J. Zuurbier, Benedikt Preckel, Nina C. Weber

**Affiliations:** 1 Laboratory of Experimental Intensive Care and Anesthesiology (L.E.I.C.A.), Anesthesiology, Amsterdam Cardiovascular Sciences, Amsterdam UMC, University of Amsterdam, 1105 AZ Amsterdam, The Netherlands; x.li1@amsterdamumc.nl (X.L.); g.romer@amsterdamumc.nl (G.R.); r.p.kerindongo@amsterdamumc.nl (R.P.K.); j.hermanides@amsterdamumc.nl (J.H.); m.w.hollmann@amsterdamumc.nl (M.W.H.); c.j.zuurbier@amsterdamumc.nl (C.J.Z.); b.preckel@amsterdamumc.nl (B.P.); 2 Department of Anesthesiology and Intensive Care Medicine, Universitätsklinikum Schleswig-Holstein, Campus Kiel, 24105 Kiel, Germany; Martin.Albrecht@uksh.de

**Keywords:** sodium glucose co-transporter 2 inhibitors (SGLT-2i’s), cell permeability, reactive oxygen species (ROS), sodium-hydrogen exchanger 1 (NHE1), NADPH oxidases (NOXs)

## Abstract

SGLT-2i’s exert direct anti-inflammatory and anti-oxidative effects on resting endothelial cells. However, endothelial cells are constantly exposed to mechanical forces such as cyclic stretch. Enhanced stretch increases the production of reactive oxygen species (ROS) and thereby impairs endothelial barrier function. We hypothesized that the SGLT-2i’s empagliflozin (EMPA), dapagliflozin (DAPA) and canagliflozin (CANA) exert an anti-oxidative effect and alleviate cyclic stretch-induced endothelial permeability in human coronary artery endothelial cells (HCAECs). HCAECs were pre-incubated with one of the SGLT-2i’s (1 µM EMPA, 1 µM DAPA and 3 µM CANA) for 2 h, followed by 10% stretch for 24 h. HCAECs exposed to 5% stretch were considered as control. Involvement of ROS was measured using N-acetyl-l-cysteine (NAC). The sodium-hydrogen exchanger 1 (NHE1) and NADPH oxidases (NOXs) were inhibited by cariporide, or GKT136901, respectively. Cell permeability and ROS were investigated by fluorescence intensity imaging. Cell permeability and ROS production were increased by 10% stretch; EMPA, DAPA and CANA decreased this effect significantly. Cariporide and GKT136901 inhibited stretch-induced ROS production but neither of them further reduced ROS production when combined with EMPA. SGLT-2i’s improve the barrier dysfunction of HCAECs under enhanced stretch and this effect might be mediated through scavenging of ROS. Anti-oxidative effect of SGLT-2i’s might be partially mediated by inhibition of NHE1 and NOXs.

## 1. Introduction

Cardiovascular diseases are the leading cause of death and disability around the world, especially in patients with diabetes [1]. Clinical trials showed that sodium glucose co-transporter 2 inhibitors (SGLT-2i’s), a class of novel glucose lowering agents, improve cardiovascular outcomes in diabetic [2,3] and non-diabetic patients [4]. One contributing mechanism could be the direct endothelial protective effect of SGLT-2i’s. Endothelial dysfunction plays a pivotal role in the progression of heart failure (HF). Increased ROS production within endothelial cells limits nitric oxide (NO) bioavailability for adjacent cardiac myocytes and decreases protein kinase G activity, which favours cardiac hypertrophy via hypophosphorylation of titin [5]. An in vivo experiment suggested that canagliflozin (CANA) restored endothelium-dependent relaxation of pulmonary arteries in hyperglycemic mice [6]. Recently, another study showed that empagliflozin (EMPA) attenuated diastolic function in non-diabetic HF pigs via restoration of NO availability and PKG activity [7]. The improved diastolic function might explain the beneficial effect of EMPA on patients with HF [8,9]. Additionally, an in vitro study showed that EMPA ameliorated the inflammatory reaction of hyperglycemia-treated endothelial cells [10]. EMPA and dapagliflozin (DAPA) prevented inflammation induced reactive oxygen species (ROS) production in human endothelial cells [11,12]. Those experiments were performed in resting endothelial cells, while in situ endothelial cells are constantly exposed to mechanical forces, e.g., cyclic stretch caused by contraction and relaxation of arteries [13]. Physiological cyclic stretch plays a crucial role in preserving endothelial monolayer integrity [14], whereas, under pathological circumstances (e.g., hypertension), enhanced stretch induces ROS accumulation, inflammatory reactions, vascular stiffness and ultimately endothelial barrier dysfunction [15,16]. Using the Flexcell^®^ Tension Systems, in vitro stretch experiments showed that 10% cyclic stretch promoted endothelium-monocyte adhesion and disrupted the integrity of the endothelial monolayer [17,18]. In contrast, 5% stretch did not induce endothelial dysfunctions and could therefore resemble a physiological condition [17].

ROS play an essential role in regulating the vascular function and might influence endothelial barrier dysfunction [19]. Increased ROS production promoted internalization and degradation of vascular endothelial (VE)-cadherin, the major component of adherent junctions (AJs) between endothelial cells, and therefore disrupted the integrity of the endothelial monolayer and increased endothelial permeability [20,21]. In alveolar epithelial cells, enhanced stretch promoted ROS production and subsequently increased cellular permeability; scavenging ROS reverted this stretch-induced barrier dysfunction, suggesting that ROS promote the permeability of dynamic cells [22].

Based on the fact that SGLT-2i’s reduce ROS production in resting human endothelial cells [11,12], and that enhanced cyclic stretch increases oxidative stress and cell permeability of endothelial cells [19], we hypothesized that SGLT-2i’s exert an anti-oxidative effect on stretched HCAECs and thereby ameliorate stretch-induced endothelial barrier dysfunction.

## 2. Results

### 2.1. SGLT-2i’s Prevent Increased Cell Permeability and ROS Production of HCAECs Exposed to 10% Stretch

**Figure 1:**
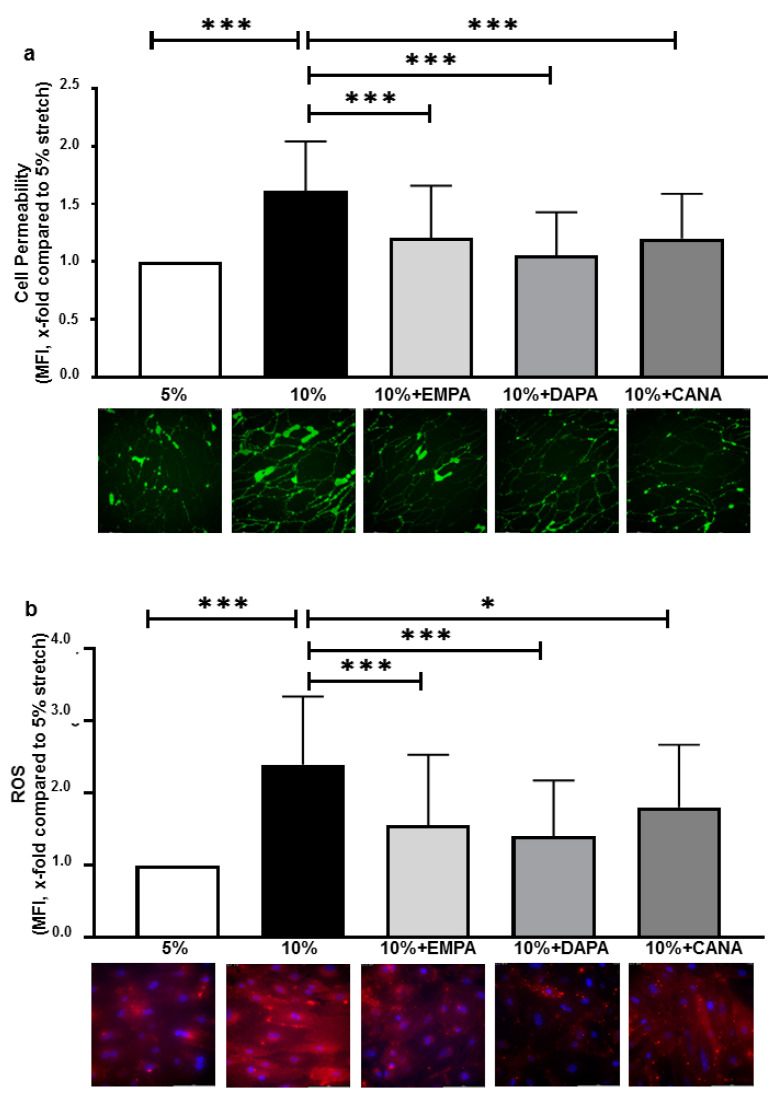
SGLT-2i’s inhibit barrier leakage and ROS production of HCAECs exposed to 10% stretch. Cells were pre-incubated for 2 h with vehicle or one of the SGLT-2i’s (1 µM EMPA, 1 µM DAPA and 3 µM CANA), and were subsequently exposed to 5% stretch plus vehicle, 10% stretch with vehicle or SGLT-2i’s for 24 h. Cell permeability was measured with live cell imaging, and 6 images were made from each condition in each individual experiment (**a**) n = 5 individual experiments). ROS levels were measured at 24 h, and six images were taken from each condition in each individual experiment (**b**) n = 5 individual experiments). Representative images are shown in the lower panel and data are presented as mean ± SD. * *p* < 0.05 vs. 10% stretch, *** *p* < 0.001 vs. 10% stretch.

Compared with 5% stretch, 10% stretch increased cell permeability 1.61 ± 0.43 fold. 1 µM EMPA, 1 µM DAPA and 3 µM CANA significantly decreased the 10% stretch-induced cell permeability (x-fold compared to 5% stretch, EMPA: 1.20 ± 0.46, DAPA: 1.06 ± 0.37, CANA: 1.20 ± 0.39, *p* all <0.001 vs. 10% stretch, Figure 1a).

Additionally, 10% stretch increased ROS production 2.39 ± 0.95 fold, 1 µM EMPA, 1 µM DAPA and 3 µM CANA significantly reduced the 10% stretch-induced ROS production (x-fold compared to 5% stretch, EMPA: 1.55 ± 0.98, DAPA: 1.41 ± 0.77, CANA: 1.80 ± 0.87, *p* all <0.05 vs. 10% stretch, Figure 1b), suggesting a class anti-oxidative effect of SGLT-2i’s.

### 2.2. SGLT-2i’s Revert Loss of VE-Cadherin Induced by 10% Stretch

**Figure 2:**
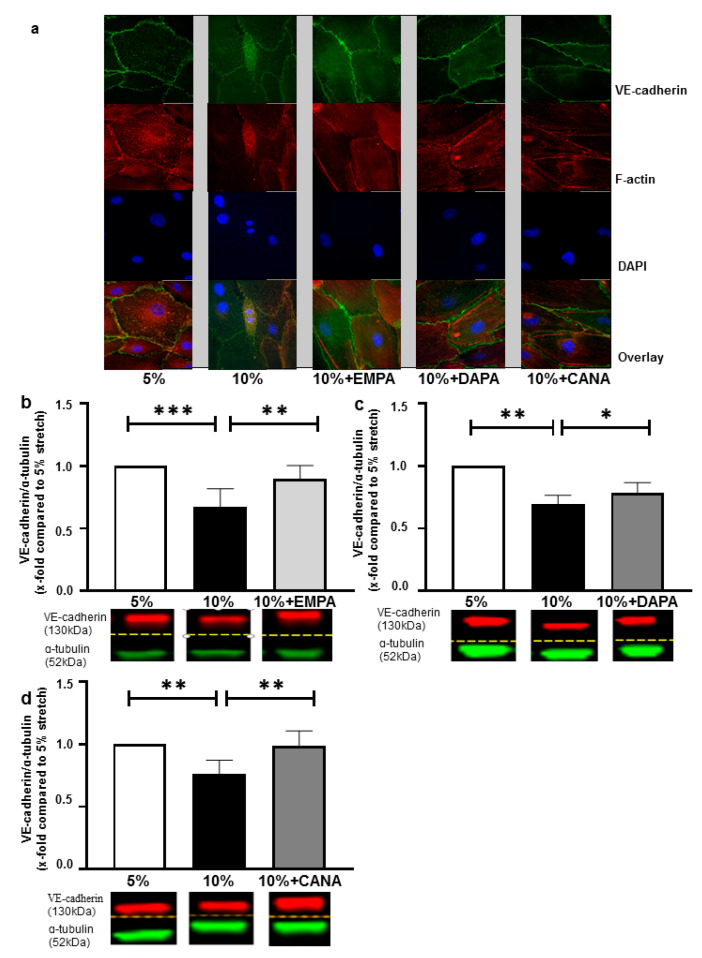
SGLT-2i’s restore VE-cadherin loss of HCAECs exposed to 10% stretch. Cells were treated with 5% stretch plus vehicle, 10% stretch plus either vehicle or one of the SGLT-2i’s (1 µM EMPA, 1 µM DAPA and 3 µM CANA). VE-cadherin and F-actin were stained after 24 h, and representative images of staining are presented (**a**) n = 3. VE-cadherin levels were quantified with western blot for EMPA treatment (**b**) n = 6, DAPA treatment (**c**) n = 6 and CANA treatment (**d**) n = 6. Representative bands of VE-cadherin and α-tubulin (internal reference) are shown in the lower panels of each bar graph (**b**–**d**) and data are presented as mean ± SD. * *p* < 0.05 vs. 10% stretch, ** *p* < 0.01 vs. 10% stretch, *** *p* < 0.001 vs. 10% stretch. Whole (uncropped) western blots can be found as supplemental information).

Fluorescence staining suggested that HCAECs exposed to 10% stretch showed elongation in morphology and loss of VE-cadherin. All three SGLT-2i’s restored VE-cadherin loss but did not attenuate altered morphology caused by 10% stretch (Figure 2a). Western Blot was carried out to confirm changes in VE-cadherin expression: EMPA, DAPA and CANA reverted stretch induced VE-cadherin degradation (x-fold compared to 5% stretch, EMPA: 0.90 ± 0.11 vs. 10% stretch: 0.67 ± 0.15, DAPA: 0.95 ± 0.09 vs. 10% stretch: 0.69 ± 0.07, CANA: 0.99 ± 0.12 vs. 10% stretch: 0.76 ± 0.11, *p* all <0.05, Figure 2b–d). None of the SGLT-2i’s influenced permeability and VE-cadherin expression of HCAECs exposed to 5% stretch (Figure S1a,b).

### 2.3. EMPA-Related Improvement of Endothelium Barrier Dysfunctions Might Be Mediated by ROS Inhibition

Previous studies showed that ROS promotes cellular permeability [23]. As part of our experiments, we also found that pyocyanin, a previously reported ROS inducer [24], increased endothelial permeability and disrupted VE-cadherin of HCAECs (Figure S3a,b).

**Figure 3:**
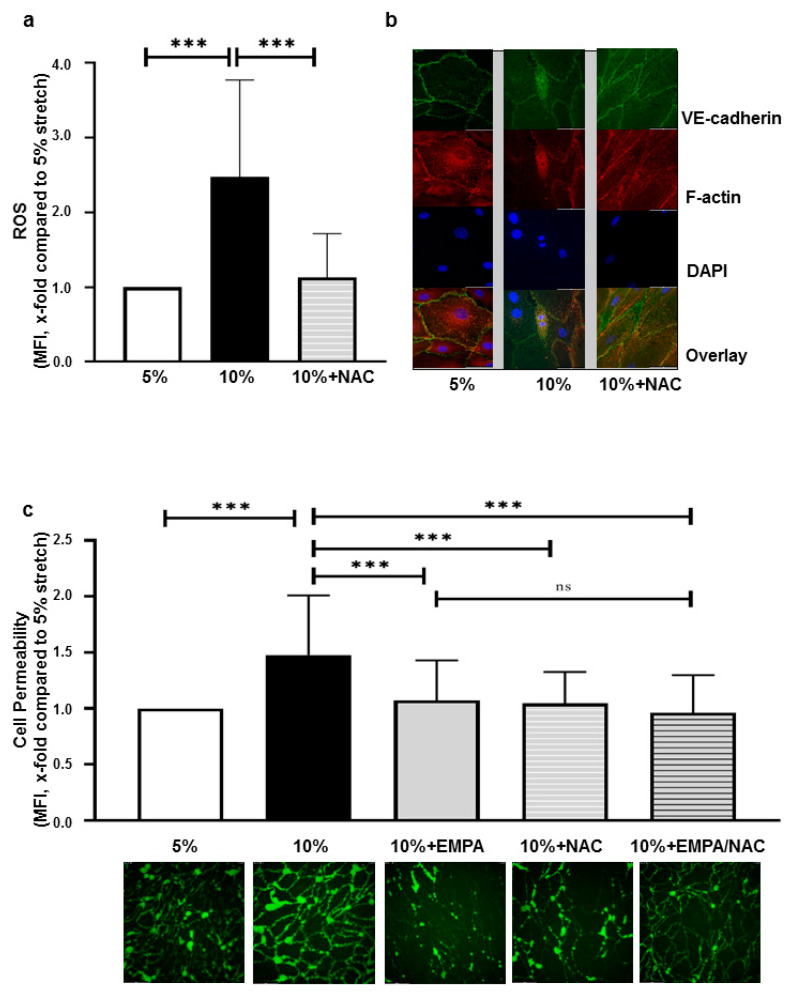
Alleviated stretch induced barrier dysfunction by EMPA might be mediated through ROS inhibition. ROS measurement and immunofluorescence staining were carried out in cells treated with 5% stretch plus vehicle, 10% stretch plus either vehicle or 5 mM NAC. After 24 h, 6 images per condition were taken from each individual experiment for ROS measurement (**a**) n = 5 individual experiments. Representative images of VE-cadherin and F-actin staining are presented in (**b**) (n = 3). Cell permeability was measured in cells treated with 5% stretch plus vehicle, 10% stretch plus vehicle, 1 µM EMPA, 5 mM NAC or combination of EMPA and NAC. Six images per condition were taken from each individual experiment (**c**, n = 5 individual experiments). Representative images of ROS and cell permeability are shown in the lower panel of each bar graph (**a**,**c**) and data are presented as mean ± SD. *** *p* < 0.001 vs. 10% stretch. ns: not significant.

NAC (5 mM) was applied on cells exposed to 10% stretch and it was found that NAC almost completely reverted the stretch induced ROS production (x-fold compared to 5% stretch, NAC: 1.13 ± 0.58 vs. 10% stretch: 2.48 ± 1.30, *p* < 0.001, Figure 3a). Fluorescence staining showed that 5 mM NAC also reverted stretch-induced VE-cadherin loss of HCAECs, without attenuating elongation of cells (Figure 3b), and NAC significantly decreased cell permeability (x-fold compared to 5% stretch, NAC: 1.04 ± 0.28 vs. 10% stretch: 1.48 ± 0.54, *p* < 0.001). Furthermore, compared with 1 µM EMPA, the combination of NAC and EMPA did not further reduce the increased cell permeability of HCAECs exposed to 10% stretch (x-fold compared to 5% stretch, EMPA+NAC: 0.96 ± 0.34 vs. EMPA alone: 1.07 ± 0.36, *p* > 0.05, Figure 3c), suggesting that the protective effect of EMPA might be mediated through ROS inhibition.

### 2.4. ROS Inhibition of EMPA Might Partly Be Mediated through Inhibiting NHE1 and NOXs

**Figure 4:**
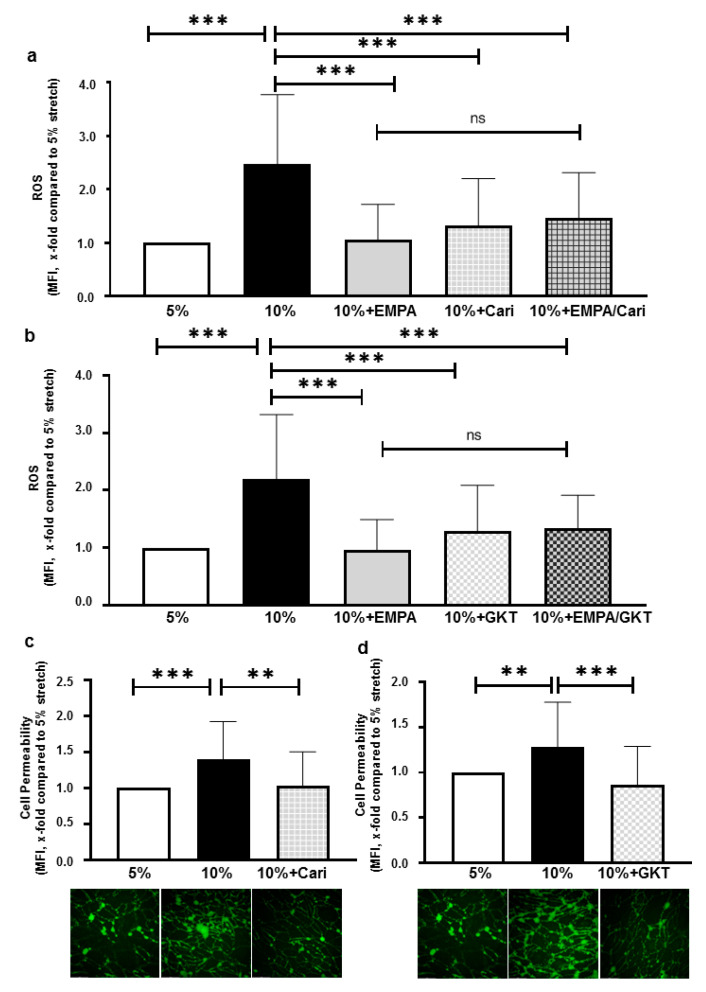
ROS inhibition by SGLT-2i’s might be mediated through inhibition of NHE1 and NOXs. ROS levels were measured in cells treated with 5%. stretch, 10% stretch plus vehicle, 1 µM EMPA, 10 µM cariporide/1 µM GKT136901 and combination of EMPA and cariporide/GKT136901 (**a**,**b**). Six images were taken from each individual experiment (**a**) n = 5 individual experiments; (**b**) n = 5 individual experiments. Cell permeability was measured in HCAECs treated with 5% stretch plus vehicle, 10% stretch plus either vehicle, 10 µM cariporide or 1 µM GKT136901 (**c**,**d**). Six images of each condition were taken from each individual experiment (**c**, n = 5 individual experiments; (**d**) n = 5 individual experiments. Representative images are shown in the lower panels and data are presented as mean ± SD. ** *p* < 0.01 vs. 10% + Vehicle, *** *p* < 0.001 vs. 10% + Vehicle. ns: not significant.

Cariporide (10 µM) significantly reduced ROS production of cells undergoing 10% stretch (x-fold compared to 5% stretch, cariporide: 1.32 ± 0.89 vs. 10% stretch: 2.48 ± 1.30, *p* < 0.001). The combination of cariporide with EMPA did not further reduce ROS production (x-fold compared to 5% stretch, EMPA+cariporide: 1.47 ± 0.85 vs. EMPA, *p* > 0.05, Figure 4a). These results correspond to the previous study showing that cariporide inhibits ROS in endothelial cells, however in that study EMPA had also an inhibitory effect on ROS largely unrelated to NHE 1 inhibition [11].

Regarding potential targets involved in ROS production, a previous study showed that enhanced cyclic stretch activated NOXs, leading to ROS production [15]. Correspondingly, 1 µM GKT136901 (a specific inhibitor for NOX1 and NOX4) significantly decreased ROS production of cells exposed to 10% stretch (x-fold compared to 5% stretch, GKT136901: 1.30 ± 0.79 vs. 10% stretch: 2.19 ± 1.12, *p* < 0.001). A combination of GKT136901 and EMPA exerted a similar inhibitory effect, comparable to EMPA alone (x-fold compared to 5% stretch, EMPA+GKT136901: 1.34 ± 0.58 vs. EMPA alone: 0.96 ± 0.54, *p* > 0.05, Figure 4b), suggesting that the inhibition of NOXs next to, or located upstream of NHE1, mediated the inhibition of ROS by EMPA in stretched endothelial cells.

Additionally, cariporide and GKT136901 both reverted the increase in cell permeability caused by 10% stretch (Figure 4c,d), further suggesting that ROS inhibition could be the mechanism behind the anti-leakage effects of SGLT-2i’s.

### 2.5. SGLT-2i’s Do Not Inhibit Interleukin Secretions of HCAECs Exposed 10% Stretch

**Figure 5:**
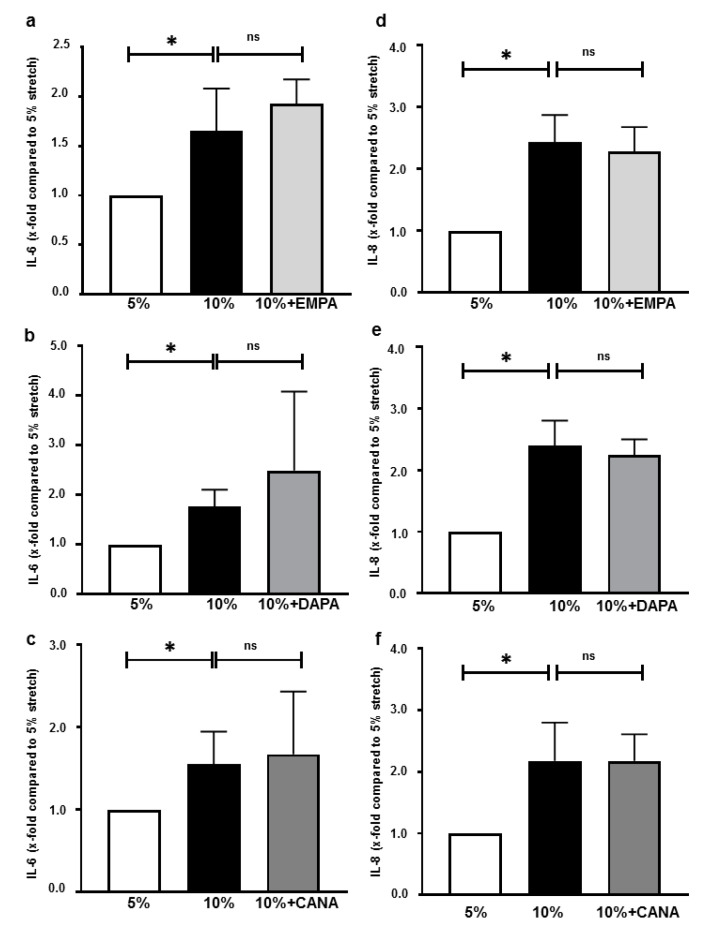
SGLT-2i’s do not inhibit interleukin secretion of endothelial cells exposed to 10% stretch. Cells were pre-incubated for 2 h with vehicle or one of SGLT-2i’s (1 µM EMPA, 1 µM DAPA and 3 µM CANA), and were subsequently exposed to 5% stretch plus vehicle, 10% stretch with vehicle or SGLT-2i’s for 24 h. IL-6 and IL-8 were measured by ELISA (**a**–**f**) n = 6. Data are presented as mean ± SD. * *p* < 0.05 vs. 10% stretch. ns: not significant.

Compared with 5% stretch, 10% stretch increased IL-6 and IL-8 secretions of HCAECs, which were not inhibited by EMPA, DAPA or CANA (Figure 5a–f).

## 3. Discussion

The major findings of the present study are: (1) SGLT-2i’s revert the increase in endothelial permeability and degradation of VE-cadherin caused by enhanced cyclic stretch; (2) this effect might be mediated through ROS inhibition; and (3) EMPA-related reduction in ROS production in stretched cells is likely mediated via inhibition of NHE1 and NOXs.

### 3.1. SGLT-2i’s Improve Stretch-Induced Endothelial Barrier Dysfunction

Enhanced stretch increased cell permeability of HCAECs and caused VE-cadherin breakdown. All three SGLT-2i’s, EMPA, DAPA and CANA reverted this stretch-induced cellular permeability and VE-cadherin degradation of HCAECs.

Previous studies suggested that endothelial cells exposed to enhanced stretch show increased permeability [25], which was partly mediated by polymerization of F-actin and disruption of VE-cadherin-mediated AJs [26]. Enhanced stretch also induced phosphorylation of VE-cadherin at Tyr658 via activating the vascular endothelial growth factor pathway, thus leading to the internalization and degradation of VE-cadherin [25]. Correspondingly, our data (Figure 2a–d) show that pathologically enhanced stretch increases endothelial permeability via VE-cadherin degradation, which is illustrated by compromised VE-cadherin mediated junctions in immunofluorescence staining and the decreased VE-cadherin expression in western blots.

### 3.2. SGLT-2i’s Inhibit ROS Production under Cyclic Stretch

In vivo data suggested that enhanced stretch induced oxidative stress in arteries [13], and in line with these previous findings we demonstrated in vitro that 10% stretch increased ROS production in HCAECs (Figure 1b), with ROS being a crucial promoter for endothelial dysfunction [23,27]. Previous data proved that EMPA and DAPA inhibited inflammation induced ROS production in resting endothelial cells [11,12], thereby restoring nitric oxide bioactivity and ameliorating endothelial dysfunctions. In the present study, we report that SGLT-2i’s attenuated oxidative stress in HCAECs exposed to enhanced stretch. 

To look into the functional link between ROS and cell permeability, we applied pyocyanin on static cells to induce ROS production [12,24]. We observed a strong increase in endothelial permeability along with F-actin polymerization and VE-cadherin disruption in cells treated with pyocyanin (Figure S3a,b). These findings indicate the role of ROS as a potential permeability triggering factor, with probably multiple pathways contributing. For instance, enhanced ROS activate Rho signaling pathways and promote the polymerization of F-actin, thus disrupting the structure of VE-cadherin mediated AJs [28]. Furthermore, ROS directly caused Src-dependent degradation of VE-cadherin from AJs, leading to impaired endothelial barrier function [29]. The scavenging ROS in our model reverted the increased ROS levels caused by 10% stretch and concomitantly alleviated the stretch induced VE-cadherin disruption and endothelial permeability. This is consistent with a previous study showing that ROS inhibition decreased stretch induced alveolar epithelial permeability [22]. Intriguingly, we found that EMPA and NAC protected the cells to a similar extent; the combination of the two agents did not further increase this effect. Taken together, these findings suggest that ROS inhibition might be a crucial mechanism by which SGLT-2i’s prevent stretch-induced endothelial permeability.

### 3.3. NHE1 and NOXs Might Be Partially Involved in the Anti-Oxidative Effect of EMPA

In order to elucidate the underlying mechanism of the anti-oxidative effect of SGLT2i’s in our model, we focused on EMPA and specifically inhibited NHE1 and NOXs with cariporide and GKT136901, respectively. Cariporide and GKT136901 almost completely blocked the stretch induced ROS production, and neither of them further reduced ROS production when combined with EMPA, suggesting that the ROS inhibition of EMPA is partially mediated by NHE1 and NOXs.

Previous studies of our group suggested the cardiovascular protective effects of SGLT-2i’s were partly mediated via NHE1 inhibition [30,31,32]. SGLT-2i’s inhibited NHE1 activation and sodium influx in isolated mice hearts, thus inducing coronary vasodilation. Additionally, stretch directly activated NHE1 of cardiac myocytes, in turn promoting a calcium influx and increasing heart contraction force [33]. DAPA reduced NHE1 expression in cardiomyocytes, thus lowering intracellular sodium concentration and improving myocardial function of diabetic mice [34]. Juni et al. also found that cariporide reduced ROS production of endothelial cells by 38%, however they also found that EMPA could reduce ROS even more in the presence of cariporide suggesting an effect of EMPA unrelated to NHE1 [11]. Another study showed that cariporide attenuated homocysteine induced endothelial dysfunction via reducing oxidative stress and inflammatory reactions [35]. In line with these studies we here prove that NHE1 activation is involved in stretch-induced ROS production of HCAECs and that the anti-oxidative effect of EMPA might be partly mediated via NHE1 inhibition. However, there is an ongoing discussion about the role of NHE1 activity in the cardiovascular protection of SGLT-2i’s. Chung and his colleagues reported a neutral effect of EMPA on NHE1 activity with isolated rat myocytes [36], which is contrary with a more recent study conducted by Zuurbier et al. [37].

Another source of ROS in blood vessels are NOXs; previous studies showed that enhanced cyclic stretch significantly increased expression levels of NOX2 and NOX4, and apocynin (a specific NOX2 blocker) reverted this effect in endothelial cells [13,38]. In mice, EMPA reverted upregulation of NOX2 and NOX4 and attenuated ischemia-reperfusion injury in the kidneys, suggesting inhibition of NOXs might be another mechanism for ROS inhibition of EMPA [39].

Interestingly, GKT136901 and cariporide exerted similar anti-oxidative effects on stretched HCAECs in our study. Several studies revealed the potential interactions between NOXs and NHE1 in oxidative stress of endothelial cells. Activated NOXs produced hydrogen peroxide, a type of stable ROS that maintains the opening of mitochondrial ATP-dependent potassium channels (mitoKATP) and thus enhances the production of mitochondrial ROS by the electron transport chain [40,41]. Mitochondrial ROS are then released to cytosol and in turn activates NHE-1 and sodium–calcium exchange, promoting influx of calcium into the cytosol [42]. Finally, increased cytosolic calcium enhances mitochondrial calcium load and further promotes ROS production [43].

Taken together, our data support that NOXs and NHE1 might contribute to the enhanced ROS production in stretched HCAECs, but how SGLT-2i’s interrupt this signaling cascade is still unclear and warrants further investigations.

### 3.4. SGLT-2i’s Do Not Alter Interleukin Secretion of Stretched Cells

In vivo and in vitro studies have shown that pathological mechanical forces stimulate cytokine production and expression of adhesion molecules, leading to stiffness of vessels and monocyte adhesion [17,44]. Interestingly, none of the three SGLT-2i’s inhibited stretch-induced interleukin secretion (Figure 5), which is in contrast to previous data showing that SGLT-2i’s inhibit interleukin production of endothelial cells from humans, as well as from *ApoE*(-/-) mice [45,46]. One potential explanation might be that the stretch-induced interleukin secretion of HCAECs is much milder than that caused by lipopolysaccharide (1.5–2 fold vs. 6–7 fold) [46], making it a less optimal model to investigate the potential anti-inflammatory effects of SGLT-2i’s.

### 3.5. Limitation and Conclusions

This study was only performed in HCAECs from non-diabetic donors and further research is required to test the effects of SGLT-2i’s in diabetic cells. For the underlying mechanisms of ROS inhibition by SGLT-2i’s, we indirectly demonstrated that inhibition of NHE1 and NOXs is involved, and how they interact in stretch-induced oxidative stress is yet unknown. Another drawback of this study is that we did not look into the involvement of SGLT-2 in the observed effects. However, the expression of *SGLT-2* in human ECs is highly questionable. Using western blot, we recently reported a potential signaling of *SGLT-2* in HCAECs, but as the bands persisted existing after the target gene was silenced at mRNA level, we doubted the specificity of these bands and performed qPCR targeting SGLT2 in HCAEC showing no detectable levels of *SGLT2* [12]. In animal endothelial cells however, the presence of SGLT-2 within has been recently proven [10], but these findings might not be comparable to our data because of the different species. Moreover, investigations into other mechanisms underlying SGLT-2i’s anti-oxidative effect are needed. For instance, EMPA might attenuate the overproduction of ROS in endothelial cells via ameliorating mitochondrial injury [11]. Another study suggested that the beneficial effect of EMPA on mitochondria was mediated via the activation of adenosine monophosphate-activated protein kinase AMPK [47]. However, the involvement of AMPK phosphorylation is still under discussion. In the previous studies of Uthman et al., AMPK activation was not observed in EMPA or DAPA, but only in CANA treated cells [46].

In conclusion, SGLT-2i’s protect human endothelial cells against cyclic stretch-induced increased permeability and this effect is most likely mediated through ROS inhibition. Our observations provide novel insights into the possible mechanisms of the cardiovascular protection of SGLT-2i’s on mechanically stimulated cells, thus helping to improve treatment of endothelial dysfunctions induced by disturbed mechanical forces.

## 4. Materials and Methods

### 4.1. Cell Culture and Cyclic Stretch Protocol

HCAECs (PCS-100-020 from ATCC, Manassas, VA, USA; C-12221 from PromoCell, Heidelberg, Germany) were used for cell culture with vascular cell growth medium (ATCC) containing supplements as previously described [46].

**Figure A1:**
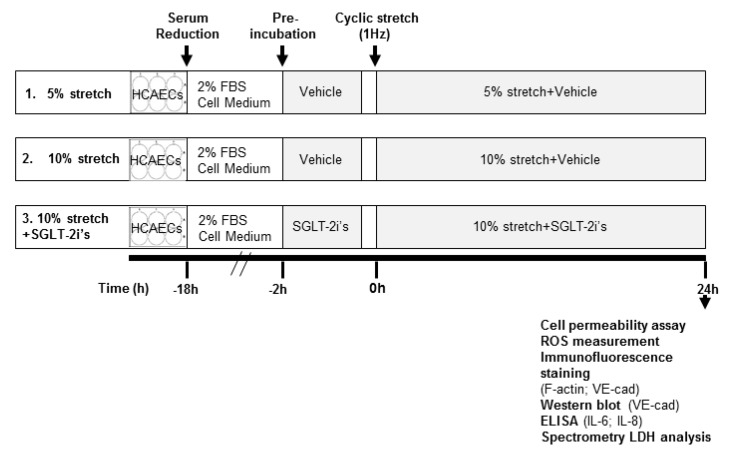
Study design Cells were starved for 18 h by serum reduction before a 2 h pre-incubation with either vehicle (0.06% DMSO) or one of the SGLT-2i’s (1 µM EMPA, 1 µM DAPA and 3 µM CANA) under starving conditions, followed by stimulation with 10% stretch for 24 h. HCAECs exposed to 5% stretch with vehicle were considered as control. Cell permeability, ROS levels, cell morphology, VE-cadherin expression, interleukin production and LDH activity were measured after 24 h.

HCAECs were seeded onto BioFlex^®^ 6-well plates (Flexcell International, McKeesport, PA, USA) at a density of 10,000–20,000 cells/cm^2^, and all plates were pre-coated overnight with 0.25 mg/mL gelatine dissolved in 0.1 M sodium bicarbonate buffer (NaHCO3, pH = 8.3). All experiments were performed with cells at passage 7 when they reached 90–100% confluency. Cyclic stretch was applied using the FX-5000T Flexcell Tension Plus system (Flexcell International, McKeesport, PA, USA). Based on previous in vitro studies [17,18], 5% stretch was considered as the physiological model and 10% as the pathological condition. HCAECs were subjected to stretch for 24 h at a frequency of 1 Hz. Cells were starved for 18 h before experiments and pre-incubated for 2 h with vehicle or SGLT-2i’s (1 µM EMPA, 1 µM DAPA and 3 µM CANA, all from MedChem, Sollentuna, Sweden). The schematic outline of this study is shown in Figure A1.

To elucidate involved mechanisms, 5 mM N-acetyl-l-cysteine (NAC, Merck Millipore, Watford, England) was applied to scavenge ROS, 1 µM GKT136901 (Calbiochem, San Diego, CA, USA) to inhibit nicotinamide adenine dinucleotide phosphate oxidases (NOXs), and 10 µM cariporide (Sigma Aldrich, St Louis, MO, USA) to inhibit sodium-hydrogen exchanger 1 (NHE1).

### 4.2. Cell Permeability Assay

A cell permeability assessment was performed using the method described by Dubrovskyi et al., which is based on high affinity binding between avidin and biotin [16].

EZ-Link NHS-LC-LC-Biotin (Thermo Fisher Scientific, Waltham, MA, USA) was added to 10 mg/mL of gelatin solution (dissolved in 0.1 M NaHCO3 buffer), to generate a solution of 0.57 mg/mL biotin and 9 mg/mL gelatin. The biotinylation of gelatin was performed at room temperature for 1h with constant stirring, and biotinylated gelatin was than aliquoted and stored at −20°C. For coating, gelatin aliquots were diluted in 0.1 M NaHCO3 buffer to generate a working solution with 0.25 mg/mL biotinylated gelatin and sterilized by a filter with a pore size of 0.2 µm (VWR international, Radnor, PA, USA). Three ml working solution were added to each well on the Bioflex plate. Plates were covered with aluminium foil and stored at 4 °C overnight, and unabsorbed protein was removed by washing twice with phosphate buffered saline (PBS, pH = 7.4).

After stretch, fluorescein isothiocyanate (FITC) labeled avidin (Thermo Fisher Scientific, Waltham, MA, USA) was added to medium (working concentration, 25 µg/mL) and incubated for 3 min, after which excessive avidin was removed by PBS washing. The plastic membranes of BioFlex plates with cells were loaded on glass cover slides with mounting fluid (Thermo Fisher Scientific, Waltham, MA, USA) containing 4’,6-diamidino-2-phenylindole (DAPI). Membranes were imaged with Leica DMi8 Advanced Light Microscopy (Leica Microsystems, Wetzlar, Germany) at 200× magnification, and six pictures of each condition were made from each individual experiment. The fluorescence was quantified using Image J1.8.0 and divided by the cell number to generate a mean fluorescence intensity (MFI). To reduce any bias, the quantification was carried out by two independent investigators (X.L. & G.R.) in a blinded manner.

### 4.3. ROS Measurement

After 24 h of cyclic stretch, CellROX ^®^ Deep Red Reagent (Thermo Fisher Scientific, Waltham, MA, USA) was added on each well to generate a working concentration of 5 µM, and plates were covered with aluminium foil and incubated at 37 °C in a humidified incubator for 30 min. Cells were than washed once with pre-warmed PBS and fixed with 4% formalin for 15 min. The plastic membranes of BioFlex plates were loaded on glass cover slides with mounting fluid containing DAPI. Membranes were imaged with Leica DM6 Wide-Field Microscopy (Leica Microsystems, Wetzlar, Germany) at 400× magnification, and 6 pictures of each condition were taken from each individual experiment. The fluorescence was quantified using Image J1.8.0 and divided by cell number to generate a MFI. To reduce any bias, the quantification was carried out by two independent investigators (X.L. & G.R.) in a blinded manner.

### 4.4. Immunofluorescence Staining

After 24 h of stretch, monolayers of HCAECs were fixed with 4% formalin for 10 min. Cells were washed three times with PBS and blocked for 30 min with 1% bovine serum albumin (PAA Cell Culture Company, Cambridge, UK). VE-cadherin monoclonal antibody (1:60 dilution, Thermo Fisher Scientific, Waltham, MA, USA) was added to each well and incubated at room temperature for 1h, unbound antibody was removed by PBS washing. Afterwards, goat anti-mouse secondary antibody (1:400, Alexa Flour^®^ 488, Thermo Fisher Scientific, Waltham, MA, USA) and Rhodamine Phalloidin (1:40, Thermo Fisher Scientific, Waltham, MA, USA) was added to each well for 30 min, followed by three times washing with PBS. Membranes were loaded on glass cover slides with mounting fluid containing DAPI and imaged with Leica DM6 Wide-Field Microscopy at 1000× magnification.

### 4.5. Infrared Western Blot

Whole cell lysates from two wells with the same treatments were combined. After 24 h of stretch, cells were washed with ice-cold PBS and collected in a lysis buffer, made from a homogenisation buffer (0.25 M sucrose and 0.02 M HEPES, pH = 7.4) with 0.5% Triton-100X, 1 mM dithiothreitol and HaltTM Protease and Phosphatase Inhibitor Single-Use Cocktail (diluted 1:100 Thermo Fisher Scientific, Waltham, MA, USA). The total protein concentration was determined by the Lowry method [48] and adjusted to 1000 µg/mL per blot. Membranes were incubated with primary antibodies against VE-cadherin (1:1000, CST, Danvers, MA, USA) and α-tubulin (1:20,000, Thermo Fisher Scientific, Waltham, MA, USA) overnight, followed by three times washing with PBS containing 0.1% tween 20 and incubation with the complementary secondary antibodies (1:5000, Li-Cor, Lincoln, NE, USA) for 1 h. The membranes were scanned with the Odyssey CLx operator (Li-Cor, Lincoln, NE, USA) after final washing, and quantifications of the signals of each band were carried out using Image StudioTM Software (Version 5.2, Li-Cor, Lincoln, NE, USA).

### 4.6. Enzyme-Linked Immunosorbent Assay (ELISA)

Two wells of supernatant from cell culture were combined and centrifuged at 4 °C for 10 min and with a centrifuge force of 150 g. Interleukin-6 (IL-6) and interleukin-8 (IL-8) in the supernatant were measured with ELISA kits (R&D Systems, Minneapolis, MN, USA) according to manufacturer’s instructions.

### 4.7. Lactate Dehydrogenase (LDH) Activity

To determine cell death, activity of LDH in supernatant was detected photospectrometrically at 25 °C, with pyruvate and nicotinamide adenine dinucleotide hydrogen (NADH). The formation rate of NAD+ from NADH was determined over 240 s and used for the measurement of total LDH activity. Compared with 5% stretch, 10% did not increase LDH activity of HCAECs, suggesting that apoptosis might not be involved in stretch induced permeability (Figure S2).

### 4.8. Sample Size and Statistical Analysis

Sample size estimation revealed that at least 5 independent experiments in each group are required to reach a power of 80%, given a physiologically relevant difference of 25% between control and intervention group and an α of 0.05 (10% standard deviation).

Statistical analyses were performed using GraphPad Prism 8.3.0 (GraphPad, San Diego, CA, USA). All data were normalized to 5% stretch and are presented as mean ± standard deviation (SD). One-way ANOVA with Bonferroni post hoc correction was carried out when comparing the difference between cells treated with SGLT-2i’s and 10% stretch. *p* ≤ 0.05, referred as *; ** for *p* ≤ 0.01 and *** for *p* ≤ 0.001, were considered statistically significant.

## Data Availability

All data generated and analyzed during the study are included in this manuscript and its supplementary information or can be obtained from the authors upon reasonable request.

## Supplementary Materials

The following are available online at https://www.mdpi.com/article/10.3390/ijms22116044/s1.
