# Supplementary material for: Sodium Glucose Co-Transporter 2 Inhibitors Ameliorate Endothelium Barrier Dysfunction Induced by Cyclic Stretch through Inhibition of Reactive Oxygen Species
Source: Int J Mol Sci. 2021 Jun 3;22(11):6044. doi: 10.3390/ijms22116044 (PMC8199893; doi:10.3390/ijms22116044)
Supplement: Supplementary file 1 [file ijms-22-06044-s001.zip › ijms-1231112-supplementary.pdf]

Supplementary Materials

Sodium glucose co-transporter 2 inhibitors ameliorate endothelium barrier dysfunction induced by cyclic stretch through inhibition of oxygen species

International Journal of Molecular Sciences

Xiaoling Li[1], Gregor Römer[1,2], Raphaela P. Kerindongo[1], Jeroen Hermanides[1], Martin Albrecht[2], Markus W. Hollmann[1], Coert J. Zuurbier[1], Benedikt Preckel[1], Nina C. Weber[1*]

[1]Amsterdam UMC, University of Amsterdam, Anesthesiology, Laboratory of Experimental Intensive Care and Anesthesiology, Amsterdam Cardiovascular Sciences, Amsterdam, The Netherlands
[2]Department of Anesthesiology and Intensive Care Medicine, Universitätsklinikum Schleswig-Holstein, Campus Kiel, Germany

* Corresponding author: Nina C Weber, PhD
Department of Anesthesiology, Laboratory of Experimental Intensive Care and Anesthesiology (L.E.I.C.A.)
Amsterdam UMC, location Academic Medical Centre (AMC)
Address: Meibergdreef 9, Room M0-128
Amsterdam, Noord-Holland, 1105 AZ, The Netherlands
Tel.: +31 20 5668215
E-mail: N.C.Hauck@amsterdamumc.nl

Figure S1
Cell permeability and VE-cadherin expression of cells under 5% stretch:

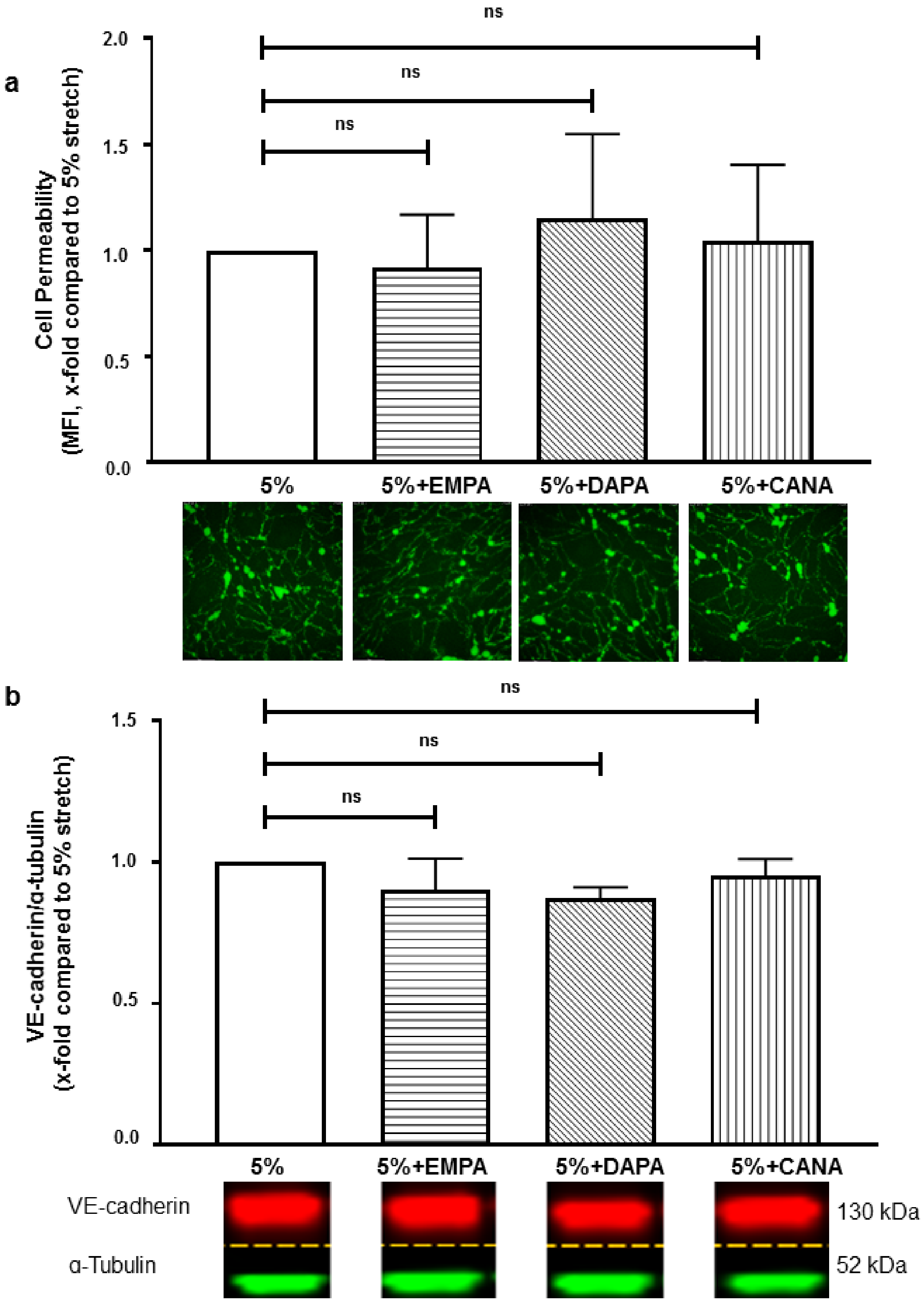

Figure S1. SGLT-2i's do not influence the barrier function of HCACEs exposed to 5% stretch. Cells were starved for 18 h by serum reduction before a 2 h pre-incubation with either vehicle (0.06% DMSO) or one SGLT-2i's (1 µM EMPA, 1 µM DAPA and 3

μM CANA), followed by exposure to 5% stretch. Cell permeability and VE-cadherin expression were measured after 24 h. Cell permeability was measured by live cell imaging, and 6 images were taken from each condition in each individual experiments (a, n=5). VE-cadherin levels were quantified with western blot (b, n=4). Representative images and bands are shown in the lower panels and data are presented as mean±SD.

Figure S2
Cell death measurement

Pyocyanin is a bacterial product from Pseudomonas aeruginosa that induces ROS production and apoptosis of cells (Manago *et al.*, 2013). HCAECs were seeded on 6-well plates at a density of 20000 cells/cm^2, and 50 μM pyocyanin was applied for 24 h to induce cell death as positive control. Two wells of the supernatant from cell cultures were combined and centrifuged at 4°C, 10 minutes at 150 g, and stored in -20°C for further analyses.

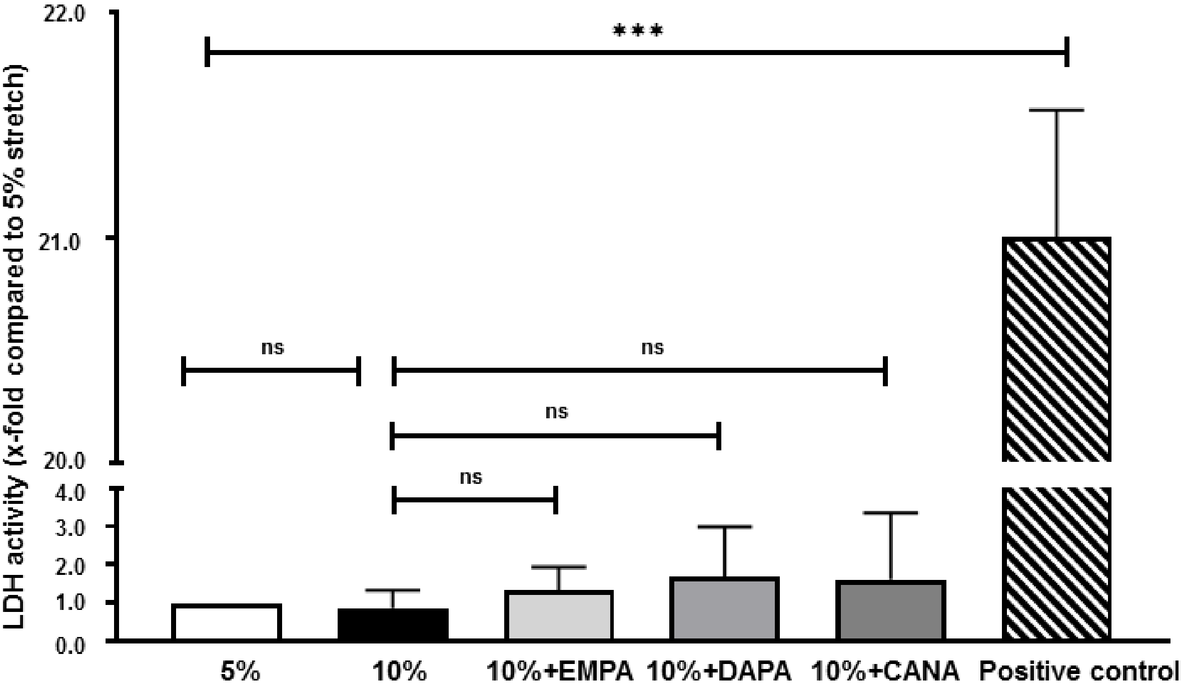

Figure S2. 10% stretch does not increase LDH activity of HCAECs. Cells were pre-incubated for 2 h with vehicle or one of the SGLT-2i's (1 μM EMPA, 1 μM DAPA and 3 μM CANA), and were subsequently exposed to 5% stretch plus vehicle, 10% stretch with vehicle or SGLT-2i's for 24 h. Cells treated with 50 μM pyocyanin for 24 h were used as positive control. LDH activity was measured photospectrometrically (n=6), and data are presented as mean±SD. ***P<0.001 *vs.* 5% stretch.

Figure S3

Cell permeability measurement with pyocyanin

ROS was induced using pyocyanin following our previous protocol (Uthman *et al.*, 2019). Briefly, cells were seeded on 12-well plates with a density of 10000 cells/cm^2 and incubated with 200 μM pyocyanin for 30 min. Cell permeability assays and staining were performed after 6 h.

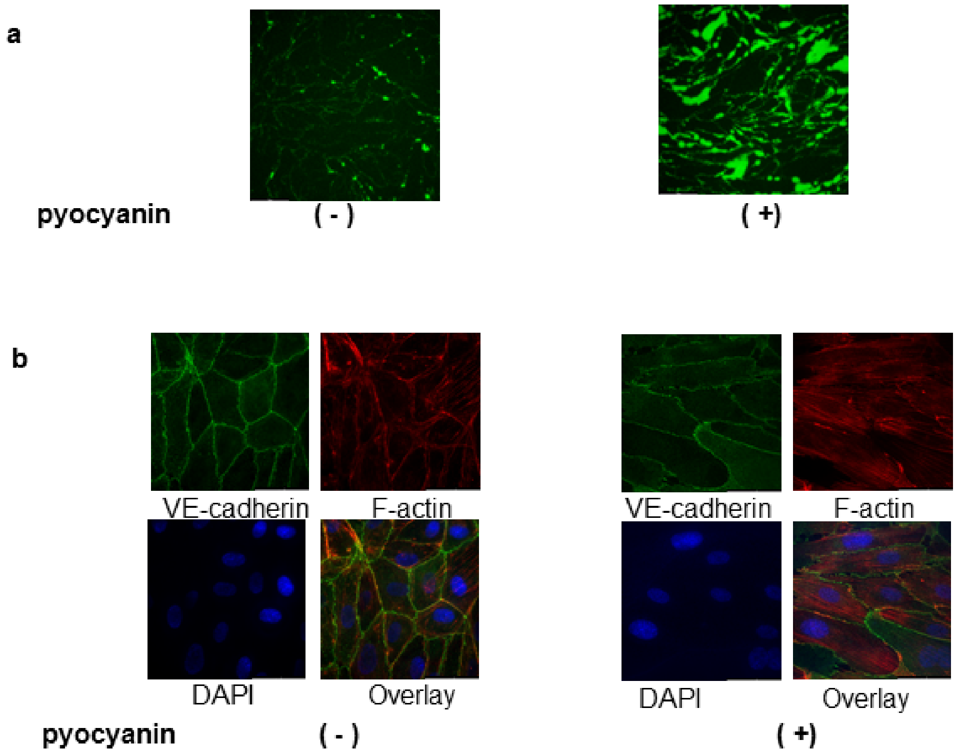

Figure S3. 200 μM pyocaynin impairs endothelium barrier functions of HCAECs. Cells were treated with 200 μM pyocyanin for 30 min. Remaining pyocyanin was removed by PBS washing and cells were incubated for 6h with fresh medium. Cell permeability was visualized with live cell imaging (a, n=5). VE-cadherin expression and F-actin arrangement were visualized with immunofluorescence staining (b, n=3). Representative images are shown.

References

1. Manago A, Becker K A, Carpinteiro A, Wilker B, Soddemann M, Seitz A P, Edwards M J, et al. (2015). Pseudomonas aeruginosa pyocyanin induces neutrophil death via mitochondrial reactive oxygen species and mitochondrial acid sphingomyelinase. Antioxid Redox Signal, 22(13), 1097-110. http://doi.org/10.1089/ars.2014.5979
2. Uthman L, Homayr A, Juni R P, Spin E L, Kerindongo R, Boomsma M, Hollmann M W, et al. (2019). Empagliflozin and Dapagliflozin Reduce ROS Generation and Restore NO Bioavailability in Tumor Necrosis Factor alpha-Stimulated Human Coronary Arterial Endothelial Cells. Cell Physiol Biochem, 53(5), 865-886. http://doi.org/10.33594/000000178
